# Influence of the oscillometric calibration method on accuracy and precision of continuous non-invasive arterial pressure measurements using the CNAP™ device

**DOI:** 10.1186/cc13311

**Published:** 2014-03-17

**Authors:** JY Wagner, I Negulescu, M Schofthaler, A Hapfelmaier, AS Meidert, W Huber, RM Schmid, B Saugel

**Affiliations:** 1 University Medical Center Hamburg-Eppendorf, Hamburg, Germany; 2 Klinikum rechts der Isar, Technical University Munich, Germany

## Introduction

The CNAP™ system (CNSystems Medizintechnik AG, Graz, Austria) provides continuous non-invasive arterial pressure (AP) measurements based on the volume clamp method using a finger cuff. Finger AP values are calibrated to oscillometric upper-arm AP measurements. In the present study we investigated the influence of the calibration approach based on oscillometric upper-arm cuff measurements on the accuracy and precision of the CNAP™ device in comparison with invasively obtained AP measurements.

## Methods

The datasets of simultaneously recorded invasive (via arterial catheter) and non-invasive (using the CNAP™ system) AP measurements in 43 patients treated in the medical ICU of a university hospital were analyzed in this study. The following comparative analyses between the two AP measurement techniques were performed: (1) comparison of CNAP™-derived AP values with invasive AP (IAP) measurements; (2) comparison of the CNAP™ oscillometric AP values used for the calibration of finger AP with IAP measurements; and (3) computer- aided calibration (CAC) of the CNAP™ finger AP values to IAP values instead of calibration to oscillometric upper-arm AP measurements with IAP measurements.

## Results

(1) The comparison of CNAP™-derived AP values with IAP measurements revealed a mean difference (± standard deviation) for mean AP, systolic AP, and diastolic AP of +0.6 mmHg (±10 mmHg), + 11 mmHg (±17 mmHg), and -6 mmHg (±9 mmHg), respectively. (2) The comparison between the oscillometric AP values used for calibration of the CNAP^TM ^device and the corresponding IAP values resulted in a mean difference (± standard deviation) of -0.8 mmHg (±8 mmHg), -5 mmHg (±14 mmHg), and +10 mmHg (±9 mmHg), respectively. (3) CAC of the CNAP^TM ^finger AP values to IAP values instead of calibration to oscillometric upper-arm AP measurements resulted in a mean difference (± standard deviation) of +4 mmHg (±8 mmHg), +5.5 mmHg (±14 mmHg), and +3 mmHg (±7 mmHg), respectively. The accuracy of CAC-CNAP^TM^-derived systolic and diastolic AP compared with the CNAP^TM^-derived AP calibrated to oscillometric AP was significantly higher (*P *= 0.004 and *P *< 0.001, respectively).

## Conclusion

When using the CNAP^TM ^system, calibration to oscillometric upper-arm AP values integrated into the CNAP^TM ^system is a relevant source of difference between CNAP^TM^-derived continuous non-invasive AP measurements and invasively assessed AP values.

